# A wide survey of heavy metals-induced *in-vitro* DNA replication stress characterized by rate-limited replication

**DOI:** 10.1016/j.crtox.2024.100152

**Published:** 2024-02-01

**Authors:** Qidong Ren, Xuejun Guo, Dong Yang, Chuanfang Zhao, Xiangyuan Zhang, Xinghui Xia

**Affiliations:** a Key Laboratory of Novel Food Resources Processing, Ministry of Agriculture and Rural Affairs/Key Laboratory of Agro-Products Processing Technology of Shandong Province/Institute of Agro-Food Science and Technology, Shandong Academy of Agricultural Sciences, 23788 Gongye North Road, Jinan 250100, China; b State Key Laboratory of Environment Simulation, School of Environment, Beijing Normal University, Beijing 100875, China; c Gene Engineering and Biotechnology Beijing Key Laboratory, College of Life Sciences, Beijing Normal University, Beijing 100875, China; d State Key Laboratory of Environmental Chemistry and Eco-Toxicology, Research Center for Eco-Environmental Sciences, Chinese Academy of Sciences, Beijing 100085, China

**Keywords:** Heavy metal, DNA replication stress, DNA replication rate, DNA replication-related disease

## Abstract

Heavy metals (HMs) are environmental pollutants that pose a threat to human health and have been accepted to cause various diseases, including cancer and developmental disorders. DNA replication stress has been identified to be associated with such diseases. However, the effect of HMs exclusively on DNA replication stress is still not well understood. In this study, DNA replication stress induced by thirteen HMs was assessed using a simplified *in-vitro* DNA replication model. Two parameters, *Ct_e_/Ct_c_* reflecting the cycle threshold value alteration and *K_e_/K_c_* reflecting the linear phase slope change, were calculated based on the DNA replication amplification curve to evaluate the rate of exponential and linear phases. These parameters were used to detect the replication rate reflecting *in-vitro* DNA replication stress induced by tested HMs. According to the effective concentrations and rate-limiting degree, HMs were ranked as follows: Hg, Ce > Pb > Zn > Cr > Cd > Co > Fe > Mn, Cu, Bi, Sr, Ni. Additionally, EDTA could relieve the DNA replication stress induced by some HMs. In conclusion, this study highlights the potential danger of HMs themselves on DNA replication and provides new insight into the possible links between HMs and DNA replication-related diseases.

## Highlights

• The *in-vitro* DNA replication rate was characterized using *Ct_e_/Ct_c_* and *K_e_/K_c_* which were calculated based on the amplification curve.
• *In-vitro* DNA replication stress induced by thirteen heavy metals (HMs) was evaluated using *Ct_e_/Ct_c_* and *K_e_/K_c_*.
• HMs induced *in-vitro* DNA replication stress were ranked as follows: Hg, Ce > Pb > Zn > Cr > Cd > Co > Fe > Mn, Cu, Bi, Sr, Ni.
• EDTA can potentially relieve the HMs-induced *in-vitro* DNA replication stress.

## Graphical abstract

**Figure:**
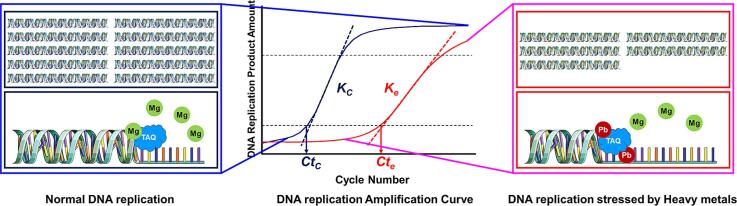

## 1 Introduction

The increased use of heavy metals (HMs) in various industrial and agricultural applications such as metal processing, mining, fertilizers, and pesticides has resulted in the accumulated metallic substances in the environment, posing a severe health hazard to humans (Briffa et al., 2020, Li et al., 2014, Qin et al., 2021). All HMs could be harmful once beyond their permissible limits in the human body, including non-essential HMs such as mercury (Hg), cadmium (Cd), and lead (Pb), and even essential HMs like copper (Cu), zinc (Zn), iron (Fe) and manganese (Mn) (Jomova et al., 2022, Sun et al., 2022). These HMs have been linked to adverse effects in humans, including cancer, neurological disorders, and growth and developmental disorders (Paithankar et al., 2021). For example, Pb, Hg, Cd, and nickel (Ni) have been classified as human carcinogens by the US EPA and the International Agency for Research on Cancer (IARC)(Tchounwou et al., 2012, Wu et al., 2016). However, the molecular mechanisms of the adverse effects induced by HMs are not yet clear, and further investigations are still required to decipher the cause-effect relationships between HMs and various diseases.

DNA replication is crucial for all organic organisms to maintain and transmit genetic material accurately (Weaver, 2011). This process is catalyzed by DNA polymerase containing Mg^2+^ in the catalytic centers (Cowan, 1998, Raper et al., 2018, Yang and Gao, 2018). Slowing or stalling DNA replication induced by various endogenous and exogenous obstacles was termed DNA replication stress, which has been identified as a significant factor in numerous diseases, such as cancer, neurological disorders, and growth and developmental disorders (Gaillard et al., 2015, Zeman and C.K. , 2014). Numerous HMs are carcinogenic, neurotoxic, and toxic to growth and development through toxicological investigations at different levels, from cellular to individual organism levels (Briffa et al., 2020, Sánchez et al., 2022, Tchounwou et al., 2012). The role of DNA replication stress in such HMs-triggered diseases has driven attention recently. The long non-coding RNA MT1DP-mediated DNA replication stress was identified to play a significant role in Cd-induced DNA damage and cancer risk using a cellular model (Feng et al., 2022). However, due to the complexity of the intracellular microenvironment, the DNA replication stress induced by HMs exclusively remains poorly understood.

To eliminate the impact of various endogenous factors, the current study was conducted to evaluate the HMs-induced DNA replication stress using a simplified *in-vitro* molecular model. The progress of this *in-vitro* DNA replication model was precisely monitored in real-time using SYBR Green II and produced an amplification curve. Two parameters were calculated by analyzing the amplification curve, which were used to reflect the HMs-induced *in-vitro* DNA replication stress characterized by a rate-limiting effect. Collectively, this study provided new insight into understanding DNA replication stress and the molecular mechanisms of DNA replication-related diseases induced by HMs.

## 2 Materials and methods

### 2.1 Materials and instruments

The constructed plasmid pET21a-CaCDC42 containing the *cdc42* gene of *Candida albicans*, which served as the template DNA, was recombined and prepared in the College of Life Science, Beijing Normal University, China. SYBR® *Premix Ex Taq*™ II containing Taq DNA polymerase catalyzing the elongation step of DNA replication, dNTPs, ROX Reference Dye II, DNA loading buffer, and SYBR Green II was purchased from Takara Bio Inc. (Dalian, China). The synthetic primers targeting *cdc42* were provided by Sangon Biotech Co. Ltd. (Beijing, China) as follows: 5′-GTGATGGTGCCGTTGGTA-3′ (forward) and 5′-CCCTGTTCCTGGGTGATT-3′ (reverse), which produced a piece of DNA containing 397 bp. Thirteen heavy metal salts, ZnCl_2_, Pb(NO_3_)_2_, CdCl_2_·2.5H_2_O, Ni(NO_3_)_2_·6H_2_O, HgCl_2_, CrCl_3_, MnCl_2_, CuSO_4_, CoCl_2_, Ce(NO_3_)_3_, Sr(NO_3_)_2_, Bi(NO_3_)_3_ and K_3_Fe(CN)_6_ were obtained from Beijing Chemical Reagent Company (Beijing, China). Ethylene diamine tetraacetic acid (EDTA), Ethidium bromide (EB), and agarose was from Sigma-Aldrich (St. Louis, USA). The DNA replication reactions *in vitro* were fulfilled and monitored using the Real-time PCR System ABI7500 (Applied Biosystems™, USA).

### 2.2 The simplified *in-vitro* DNA replication and HMs intervention

As described below, the template DNA, DNA polymerase, synthetic DNA primers, and buffer containing dNTPs compose the DNA replication reaction *in vitro*. It should be noted that the amount of each group's initial template DNA was equal in the current study. The 20 μL *in vitro* reaction system consisted of 10 μL SYBR® Premix Ex Taq™ II reaction buffer, 0.3 μL forward primer, 0.3 μL reverse primer, 0.4 μL ROX Reference Dye II, 1 μL template DNA (80 ng in each group), and 8 μL ddH_2_O (control group) or HMs solution at indicated concentrations dissolved in ddH_2_O (experimental group). EDTA at concentrations equal to corresponding HMs was added into the reaction system to assess whether HMs functioned in the ion form. The fluorescent dye SYBR Green II specific for dsDNA was applied to record the fluorescence signal, which precisely represented the amount of newly synthesized dsDNA in real-time using the Real-time PCR System ABI7500. The condition of the DNA replication reaction was set as follows: 94 ℃ for 5 min, 30 amplification cycles (94 ℃ for 30 s, 54 ℃ for 30 s, and 72 ℃ for 60 s), followed by a dissociation step. Finally, the DNA replication amplification curve was depicted based on the SYBR Green II fluorescence signal. The effects on the DNA replication rate induced by HMs could be reflected through the mathematic analysis of the DNA replication amplification curve described below. Additionally, the final synthetic DNA product of Hg, Ce, Pb, Cr, Bi, and Sr, showing different inhibitory effects on the DNA replication rate, were analyzed using agarose gel electrophoresis (AGE) to confirm the inhibitory effect and link the rate and final product amount of DNA replication affected by such HMs.After *in-vitro* DNA replication, the reaction mixture was vortexed, centrifuged, and the supernatant was collected. To prepare for gel electrophoresis, 5 μL of the supernatant was mixed with 1 μL of DNA loading buffer. The mixture was then applied to a 1 % agarose gel that contained 1 ‰ ethidium bromide (EB). The gel electrophoresis was run at 120 V for 20 min. After the electrophoresis, the band was visualized and captured using ChemiDoc™ Touch Imaging System (Bio-Rad, USA) under ultraviolet light.

### 2.3 The mathematic model analyzing the simplified *in-vitro* DNA replication

**Fig. 1:**
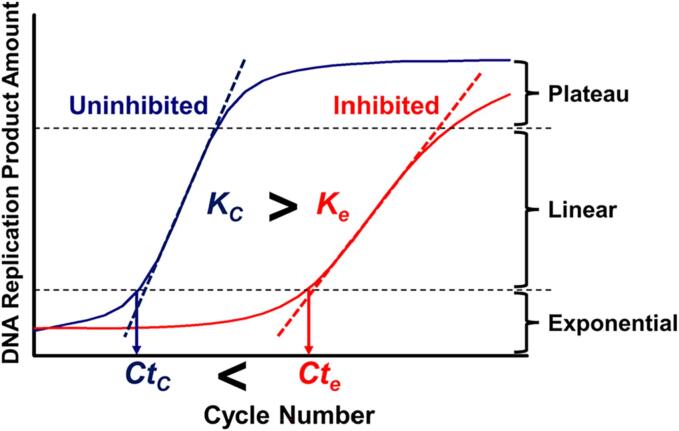
Schematic amplification curve of DNA replication process in the presence or absence of HMs. The DNA replication curve could be divided into exponential, linear, and plateau phase according to the feature of curve. *K_c_*: the linear phase slope of the control group, *Ct_c_*: the cycle threshold value (*Ct*) of the control group, *K_e_*: the linear phase slope of the HM-treated group, *Ct_e_*: the Ct of the HM-treated group.

The Real-time PCR System ABI7500 (Applied Biosystems™, USA) was used to record the newly synthesized DNA products in each cycle. An S-shaped amplification curve was formed, which had three phases: exponential, linear, and plateau (Fig. 1). The cycle threshold value (*Ct*) was automatically calculated by the Real-time PCR System and utilized to reflect the time required for DNA product accumulation to “take off” point at the junction between the exponential and linear phases. It's important to note that *Ct* did not represent the initial abundance of template DNA used in traditional qPCR analysis since the amount of each group's initial template DNA was equal. Instead, *Ct* represented the DNA replication rate at the exponential phase. The slope of the linear phase was also calculated to represent the rate of DNA replication in this phase. To assess the impact of HMs on *in-vitro* DNA replication, two parameters were calculated based on the *Ct* and *K*. The first parameter, *Ct_e_/Ct_c_*, indicating the relative *in-vitro* DNA replication rate in the exponential phase, was calculated as the ratio of *Ct* of the HMs treated group (*Ct_e_*) compared to the control group (*Ct_c_*) as shown in the formula (1). If *Ct_e_/Ct_c_* was above 1.0, it suggested that the DNA replication rate at the exponential phase decreased. The second parameter, *K_e_/K_c_*, reflecting the relative *in-vitro* DNA replication rate in the linear phase, was calculated as the ratio of *K* of the HMs treated group (*K_e_*) compared to the control group (*K_c_*) as demonstrated in the formula (2). Once *K_e_/K_c_* was below 1.0, it indicated that the rate of DNA replication in the linear phase declined.

(1) $$\frac{{\mathrm{Ct}}_{e}}{{\mathrm{Ct}}_{c}}=\frac{The\;Ct\;of\;the\;HMs-treated\;group}{The\;Ct\;of\;the\;control\;group}$$

(2) $$\frac{K_{e}}{K_{c}}=\frac{The\;linear\;phase\;slope\;of\;the\;HMs-treated\;group}{The\;linear\;phase\;slope\;of\;the\;control\;group}$$

### 2.4 Computational methods

The crystal structure of Taq DNA polymerase (PDB code: 1QTM) (Li et al., 1999) was downloaded from Protein Data Bank (www.rcsb.org). The DNA polymerase catalytic center containing two Mg^2+^ was exhibited using PyMol and MOE to depict the binding sites of Mg^2+^ under physiological conditions. Before analyzing, the structures of DNA and DNA polymerase were prepared with QuickPrep of MOE by removing water molecules, adding hydrogen atoms and minimizing residues within 8 Å from ligand. Subsequently, Hg^2+^, Pb^2+^, Cd^2+^, and Cu^2+^, representing group 1, 2, 3, and 4, respectively, was used to replace the Mg^2+^ at the catalytic center. Structures of complex with different metal ions were prepared with QuickPrep of Molecular Operating Environment (MOE), and visualized using PyMol and MOE software.

### 2.5 Statistical analysis

Values are presented as mean ± standard deviation (SD) of three independent experiments. One-way analysis of variance (ANOVA) followed by Newman-Keuls multiple comparisons test was used to determine the difference of each HM at indicated concentrations compared to the control group. Additionally, Student’s *t*-test was used to assess differences between the groups with or without EDTA in the EDTA co-treatment study. P < 0.05 was considered as statistically significant. Figure generation was conducted using Origin 8.0 software.

## 3 Results

### 3.1 Hms-induced DNA replication stress evaluated by *Ct_e_/Ct_c_*

**Fig. 2:**
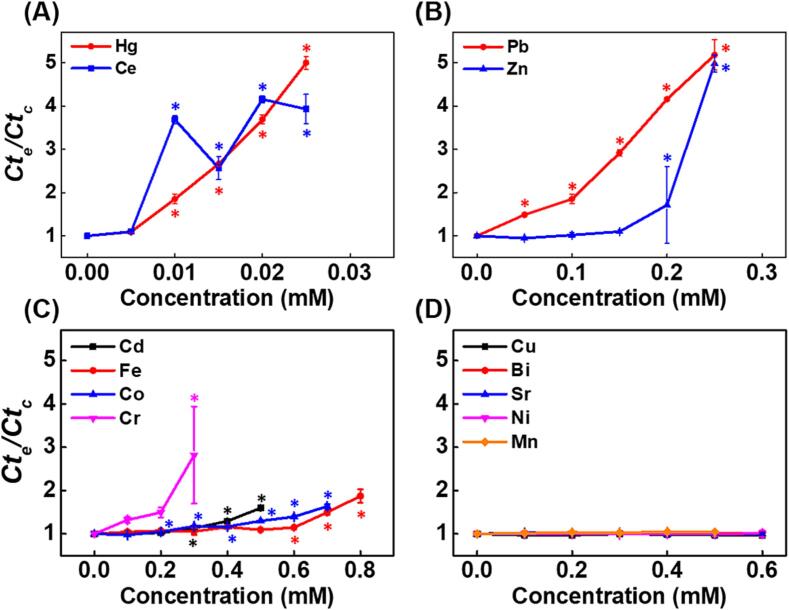
Effects of HMs on the DNA replication rate in the exponential phase assessed by *Ct_e_/Ct_c_*. (A) Hg and Ce, from 0.005 to 0.025 mM, (B) Pb and Zn, from 0.05 to 0.25 mM, (C) Cr, Cd, Fe, and Co, from 0.1 mM to indicated concentrations, (D) Mn, Cu, Bi, Sr and Ni, from 0.1 mM to indicated concentrations. Data are expressed as mean ± SD (n = 3). *p < 0.05 represents the significant difference compared with the control group.

Firstly, the impact of thirteen HMs on the *in-vitro* DNA replication rate in the exponential phase was investigated using the parameter *Ct_e_/Ct_c_*. As shown in Fig. 2A, Hg and Ce increased the *Ct_e_/Ct_c_* from 1.09 to 4.99 and 1.10 to 3.93 at concentrations from 0.005 to 0.025 mM. No fluorescent signal was obtained when the concentration of these two HMs exceeded 0.025 mM. As illustrated in Fig. 2B, the *Ct_e_/Ct_c_* rose from 1.49 to 5.18 and 1.72 to 4.98 treated with Pb and Zn at concentrations from 0.1 to 0.25 mM, respectively. In addition, the *Ct_e_/Ct_c_* also increased in the presence of Cd, Cr, Co, and Fe with higher effective concentration and weaker inhibitory effect (Fig. 2C). No fluorescent signal was detected once these three HMs exceeded the highest concentrations shown in Fig. 2A-C. As for Mn, Cu, Bi, Sr, and Ni, the *Ct_e_/Ct_c_* remained about 1.0 at concentrations from 0.1 to 0.6 mM (Fig. 2D). Collectively, according to the effective concentrations and rate-limiting effects based on the analysis of *Ct_e_/Ct_c_*, the direct HM-induced DNA replication stress in the exponential phase was ranked as follows: Hg, Ce > Pb > Zn > Cr > Cd > Co > Fe > Mn, Cu, Bi, Sr, Ni.

### 3.2 HMs-induced DNA replication stress evaluated by *K_e_/K_c_*

**Fig. 3:**
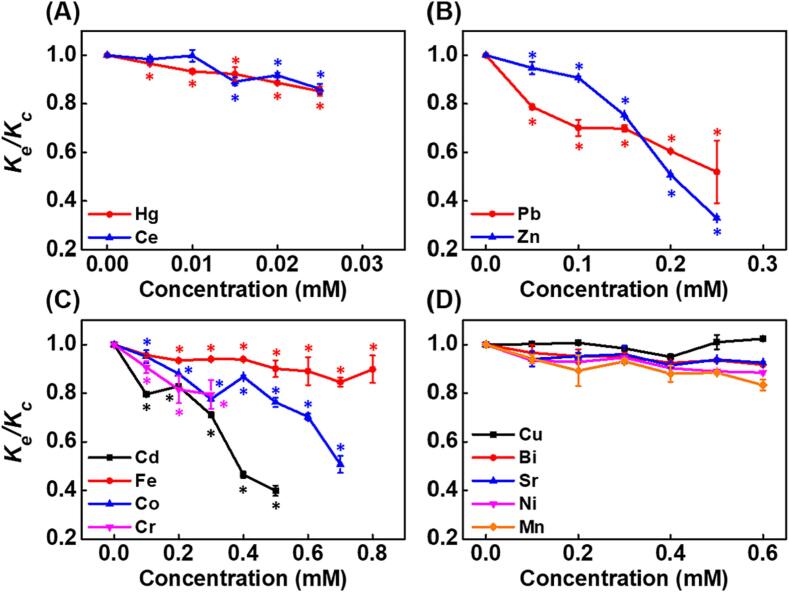
Effects of HMs on DNA replication rate in the linear phase assessed by *K_e_/K_c_*. (A) Hg and Ce, from 0.01 to 0.025 mM, (B) Pb and Zn, from 0.05 to 0.25 mM, (C) Cr, Cd, Fe, and Co, from 0.1 mM to indicated concentrations, (D) Mn, Cu, Bi, Sr, and Ni, from 0.1 mM to indicated concentrations. Data are expressed as mean ± SD (n = 3). *p < 0.05 represents the significant difference compared with the control group.

To evaluate the stresses of thirteen HMs on DNA replication in the linear phase, the parameter *K_e_/K_c_* was applied. As shown in Fig. 3A, the *K_e_/K_c_* significantly decreased from 0.93 to 0.85 and 0.99 to 0.86 in the presence of Hg and Ce at 0.005 to 0.025 mM, respectively. Following Hg and Ce, the *K_e_/K_c_* declined from 0.79 to 0.52 and 0.94 to 0.33 treated with Pb and Zn at concentrations from 0.05 to 0.25 mM, respectively (Fig. 3B). The *K_e_/K_c_* declined from 0.94 to 0.79, 0.80 to 0.40, and 0.95 to 0.51, in the presence of Cr, Cd, and Co at concentrations from 0.1 to 0.3 mM, 0.1 to 0.5 mM, and 0.1 to 0.7 mM, respectively (Fig. 3C). The *K_e_/K_c_* also declined weakly in the presence of Fe at concentrations from 0.1 to 0.8 mM (Fig. 3C). The *K_e_/K_c_* of Mn, Cu, Bi, Sr and Ni were not changed significantly at concentrations from 0.1 to indicated concentrations (Fig. 3D), though slightly decreasing effect was exhibited in the Mn, Bi, Sr, and Ni treated group. The effective concentrations and rate-limiting effects of HMs on DNA replication in the linear phase evaluated using *K_e_/K_c_* were almost consistent with the results in the exponential phase using *Ct_e_/Ct_c_*. In conclusion, the HM-induced DNA replication stress in the linear phase was ranked as follows: Hg, Ce > Pb > Zn > Cr > Cd > Co > Fe > Mn, Cu, Bi, Sr, Ni.

### 3.3 HMs-induced DNA replication stress evaluated by AGE

**Fig. 4:**
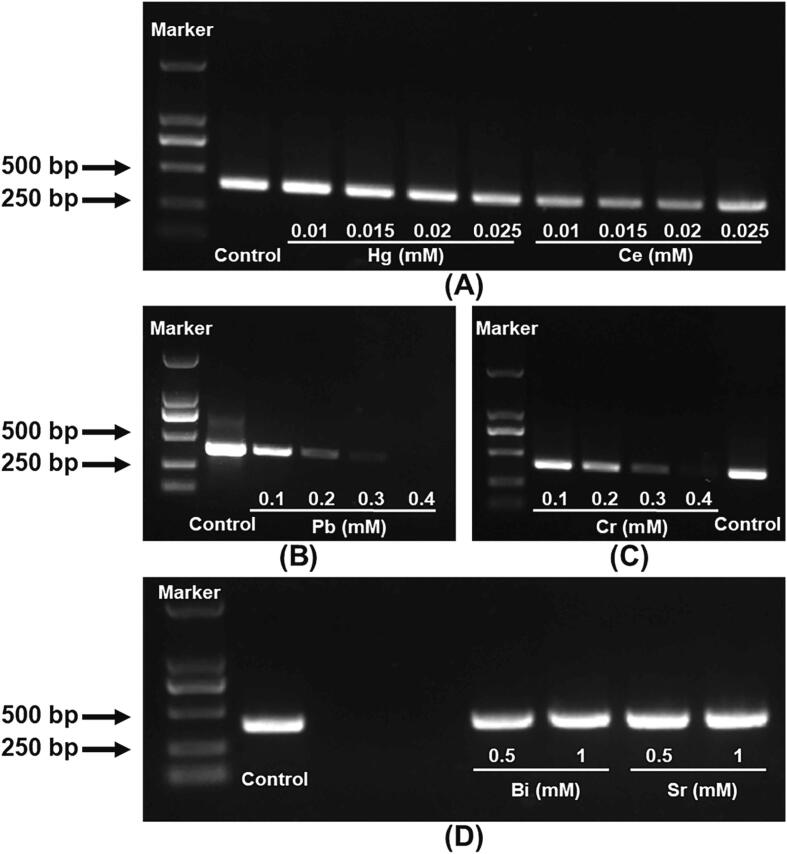
Effects of 6 HMs on DNA replication product amount assessed using AGE. (A) Hg and Ce, (B) Pb, (C) Cr, (D) Bi and Sr.

The end products of the *in-vitro* DNA replication were commonly evaluated *via* AGE. Consequently, to link the effect of HMs on the rate and the end product amount of DNA replication, the final *in-vitro* DNA replication products of Hg and Ce representing group 1, Pb representing group 2, Cr representing group 3, and Bi and Sr representing group 4 were assessed using AGE. The results showed that the concentration-dependent decline in the amount of synthetic DNA product occurred after treatment with Hg, Ce, Pb, and Cr, at concentrations ranging from 0.015 to 0.025 mM, 0.01 to 0.025 mM, 0.1 to 0.4 mM, and 0.1 to 0.4 mM, respectively (Fig. 4A-C). However, Bi and Sr did not have any effect on the yield of DNA replicated products, even at 1.0 mM (Fig. 4D). These results were consistent with those obtained through the evaluation of *Ct_e_/Ct_c_* and *K_e_/K_c_*, which showed that HMs-induced DNA replication stress characterized by a limited rate could finally lead to decreased DNA replication products.

### 3.4 HMs-induced DNA replication stress relieved by EDTA

**Fig. 5:**
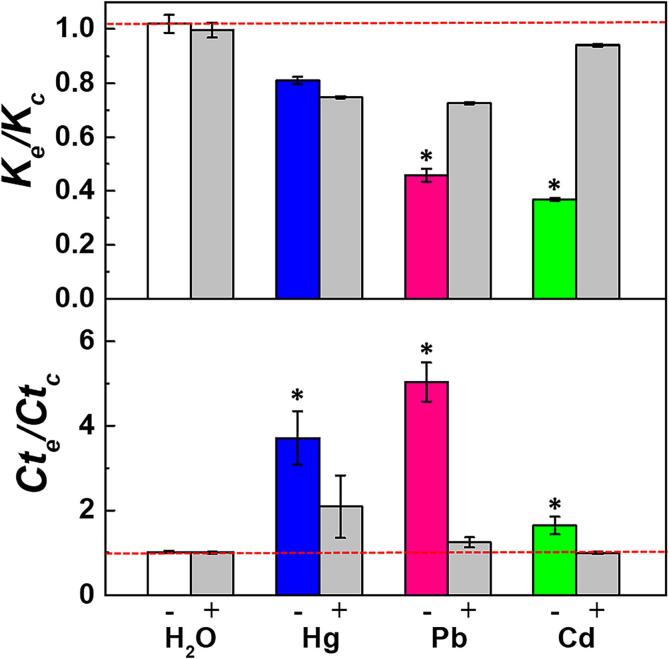
HMs-induced DNA replication stress was partly relieved by EDTA. “-”: Control or HM group in the absence of EDTA, “+”: Control or HM group in the presence of EDTA. The concentrations of Hg, Pb, and Cd was 0.025 mM, 0.2 mM, and 0.5 mM. The concentrations of EDTA were equal to corresponding HMs and the highest concentration of EDTA was 0.5 mM. *P < 0.05 represents the significant difference from the corresponding EDTA-added group.

To determine the effective form of HMs, the metal chelating agent EDTA was added into the reaction system at concentrations equal to corresponding HMs. Three HMs representing different rate-limiting effects, Hg, Pb, and Cd, were co-incubated with EDTA in the *in vitro* DNA replication system at the highest effective concentrations of each HM. As depicted in Fig. 5, the *K_e_/K_c_* was significantly increased from 0.46 and 0.37 to 0.73 and 0.9, while the *Ct_e_/Ct_c_* was reduced from 5.03 and 1.64 to 1.24 and 1.00, for Pb and Cd in the presence of EDTA. However, EDTA exhibited a limited recovery effect on the inhibitory effect of Hg, as demonstrated that the *Ct_e_/Ct_c_* weakly decreased from 3.71 to 2.09, and the *K_e_/K_c_* even decreased slightly in the presence of EDTA. However, the addition of EDTA at 0.5 mM in the control group just containing Mg^2+^ demonstrated no significant impact on the DNA replication rate according to the analysis of both *Ct_e_/Ct_c_* and *K_e_/K_c_* (Fig. 5), which indicated that the complex formed by EDTA and metal ions had no significant impact on *in-vitro* DNA replication. Collectively, these results suggested that some HMs could directly induce DNA replication stress in the ion form. However, the precise effective form of HMs still needs to be further verified.

### 3.5 Molecular simulation of HM ions interaction with Taq DNA polymerase at the catalytic center

**Fig. 6:**
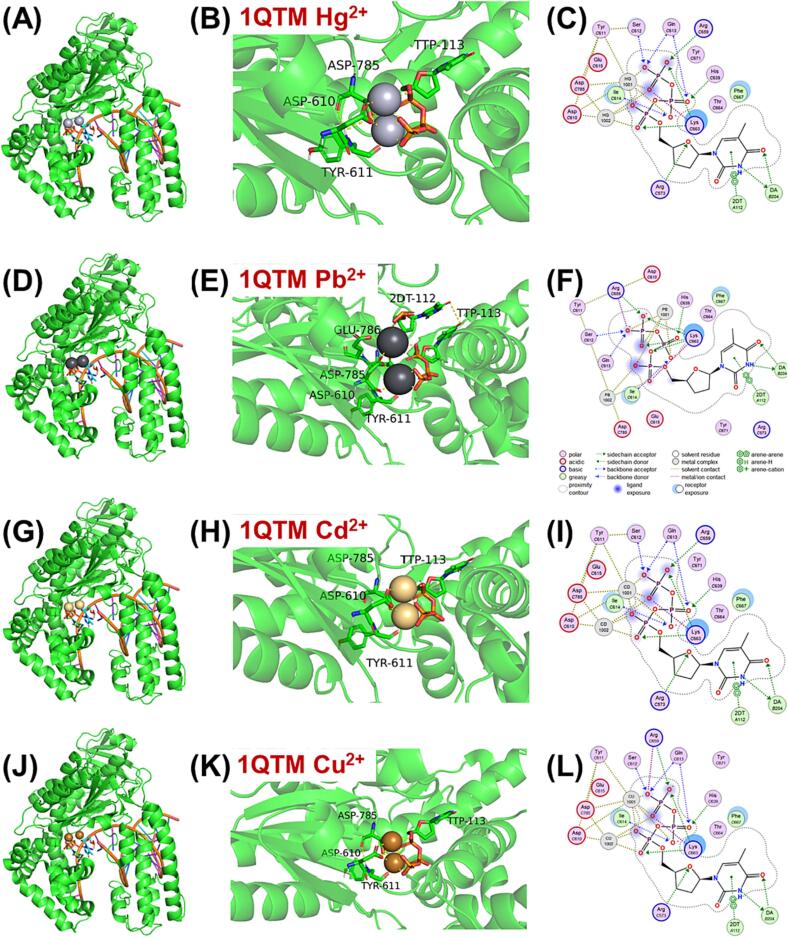
The residues binding with HM ions at the catalytic center of Taq DNA polymerase was similar to Mg^2+^. The Taq DNA polymerase catalytic center conformation and binding residues of Hg^2+^ (A)–(C), Pb^2+^ (D)–(F), Cd^2+^ (G)-(H), and Cu^2+^ (J)–(L).

Mg^2+^ has been revealed to be present at the catalytic centers of several DNA polymerases including Taq DNA polymerase, and play a significant role in DNA replication. It has been reported that Mg^2+^ forms electrostatic interactions with specific residues ASP-785, ASP-610, and TYR-611 in Taq DNA polymerase (PBD: 1QTM) (Fig. S1A) (Li et al., 1999). Mn^2+^ has also been shown to interact with Taq DNA polymerase (PBD: 6FBG) at the same residues as Mg^2+^ (Fig. S1B) (Kropp, 2018), indicating that HM ions could potentially replace Mg^2+^ at the catalytic center. To investigate the mechanisms of HMs interacting with Taq DNA polymerase, molecular simulations of Hg^2+^, Pb^2+^, Cd^2+^, and Cu^2+^, which represented HMs with different rate-limiting effects, were performed using PyMol and MOE software. The simulations showed that all four HM ions interacted with the Taq DNA polymerase catalytic center at residues ASP-785, ASP-610, and TYR-611. Pb^2+^ was found to interact with additional residues including TTP-113 and GLU-786, due to its larger radius (Fig. 6). These results suggested that the replacement of Mg^2+^ by some HMs might not alter the conformation of Taq DNA polymerase, but further verification is needed to understand the mechanisms of HM binding with Taq DNA polymerase.

## 4 Discussion

The binding of HMs with DNA polymerase has been investigated by numerous fundamental studies, demonstrated by crystal structures of various DNA polymerase containing metal ions at the active sites (Asmuss et al., 2000, Brautigam and Steitz, 1998, Kim et al., 1995, Li et al., 1998, Franklin and Steitz, 2001). It has been demonstrated that Mg^2+^ is required in the catalytic centers of all DNA polymerases and plays a vital role in efficient and faithful DNA replication (Gao and Yang, 2016, Yang and Gao, 2018, Yang et al., 2016). As Fig. S1 illustrated, two Mg^2+^ were close to the binding sites of DNA with Taq DNA polymerase (Eom et al., 1996). In the closed form of the ternary complex, one Mg^2+^ was ligated in the basal octahedral plane by four oxygen atoms, and the other Mg^2+^ was coordinated in the octahedral plane by oxygen atoms from the carboxylate of ASP-785 and the a-phosphate, while Mn^2+^ could replace one Mg^2+^ in the catalytic center of DNA polymerase (Fig. S1). In the current study, Hg^2+^, Pb^2+^, Cd^2+^, and Cu^2+^ were used to replace Mg^2+^ in this center to explore the potential interactive mechanisms of these four representative heavy metals (HMs). However, no significant changes were observed in the binding sites of HM ions and the conformation of Taq polymerase after replacing of these HMs. Additionally, the crystal structure analysis revealed that Zn^2+^ interacts with HIS-21, ASP-18, ASP-119, and ASP-142, but not with the catalytic centers of Taq DNA polymerase (PBD: 1TAU) (Fig. S2) (Eom et al., 1996). These results suggested that HMs might cause DNA replication stress by binding with the polymerase at sites rather than the catalytic centers. Nevertheless, further research is necessary to understand the binding mechanisms of HMs and DNA polymerase.

The interactions of HMs and DNA have also been extensively investigated (Duguid et al., 1995, Oliveira et al., 2008, Zhang et al., 2014). Four binding sites on dsDNA, including the oxygen atoms on the phosphates, hydroxyl groups on ribose, nitrogen atoms in the base rings, and keto groups on the exocyclic base, are easily targeted by HMs (Anastassopoulou, 2003, Koji Kawata et al., 2007). Some HMs, such as Pb^2+^, Cd^2+^, and Ni^2+^, can interact with DNA complicatedly with more than two potential binding sites including phosphate groups, purine N7 and pyrimidine N3 atoms (Anastassopoulou, 2003). The interaction of HMs and DNA could lead to DNA damage. For example, Cr^3+^ and Pb^2+^ have been reported to induce DNA damage *via* directly forming adducts with DNA (Anatoly Zhitkovich et al., 2001, Arakawa et al., 2000, Smirnov et al., 2002).

Interactions of HMs with DNA polymerases and DNA might lead to DNA replication slowing down and even stalling, which is termed DNA replication stress. However, few studies researched the HMs induced DNA replication stress at the molecular level. In 2010, Opel and colleagues conducted an assessment of the mechanism of PCR inhibition induced by seven different compounds (Opel et al., 2010). The cycle threshold value (*Ct*) and other parameters based on observation of the DNA replication amplification curves were used to evaluate the changes induced by compounds. Although they did not establish a mathematic model to quantitively analyze the DNA replication amplification curve, their study suggested that the impact of compounds on the DNA replication process, including the DNA replication rate, could be assessed through the precise multiparameter analysis of DNA replication curve.

Differing from previous investigations of simple metal/DNA and metal/DNA polymerase interactions, this current study quantitatively researched the impact of HMs on the *in-vitro* DNA replication progress containing DNA and DNA polymerase and revealed the DNA replication stress induced by HMs exclusively. Furthermore, to determine whether the interaction with Taq DNA polymerase or DNA was the driver, the impact of HMs on DNA replication could be evaluated using reaction mixtures containing a concentration gradient Taq DNA polymerase or DNA template, respectively. Melt curve analysis is commonly used to determine the alteration of the PCR product. It has been reported that the compound binds to DNA could shift the melt curve shift and alter the melting temperature (TM) (Opel et al., 2010). Therefore, melt curve analysis could also be applied to evaluate the contribution of the HMs’ interaction with DNA in the DNA replication stress induced by HMs.

The DNA replication-associated carcinogenicity and genotoxicity induced by HMs were identified predominantly through toxicological investigations at cellular, tissue, and individual organism levels phenomenally (Koji Kawata et al., 2007, Sánchez et al., 2022, Tchounwou et al., 2012). However, various cellular biological effects induced by HMs, such as oxidative stress, could also interfere with DNA replication (Andrs et al., 2023, Paithankar et al., 2021). Thus, the descriptive toxicological studies at the above-mentioned levels are difficult to get an in-depth understanding of DNA replication stress induced by HMs exclusively. To explore the DNA replication stress induced by HMs solely, the *in-vitro* DNA replication at the molecular level containing DNA template, DNA polymerase, and primers is preferable. A few studies have reported the effects of Ni, Mn, and cobalt (Co) ions on DNA replication *in vitro* by analyzing the end-point products of synthesized DNA products using AGE (Snow et al., 1993, Tabor and C.C.R. , 1989, Vashishtha and Konigsberg, 2016). In 2010, a colourimetric method was reported to roughly monitor the DNA replication progress *in vitro* by quantifying the inorganic phosphate content in the PhPPase-coupled DNA replication mixture (Lee et al., 2009, Park et al., 2010). However, the unavailability to monitor the dynamic process in real-time and low quantization accuracy limited the application of these two methods in assessing the effects of HMs on DNA replication. In the current study, the progression of *in-vitro* DNA replication was precisely monitored and quantified in real-time using SYBR Green Ⅱ, a specific fluorescent probe for DNA double-stranded (Ginzinger, 2002). The accumulation of newly synthesized DNA products under the condition of an equal initial amount of DNA template and equivalent DNA polymerase could be accurately reflected by the SYBR Green Ⅱ fluorescence signal, which could be analyzed mathematically to evaluate the effect on DNA replication of HMs.

Mercury compounds escepiallay methylmercury have been reported to impact the nervous system development of fetuses at low doses, which was strongly determined by the temporal and regional occurrence of cell proliferation and differentiation (Tatsuta et al., 2018, Zahir et al., 2005). Here, Hg-induced DNA replication stress was revealed which provided new explain of the development toxicity caused by Mercury compounds and emphasized the toxicity of Mercury ion. Similarly, Ce was found to significantly induce the DNA replication stress in this study, which might also partly account for the previous observations of the Ce-caused developmental toxicity of animal embryos (Oral et al., 2010). Though as an essential HM, Zn has been proved to drastically inhibit the catalytic activity of DNA polymerase α, possibly by perturbing metal-mediated transactions in the polymerase active site and affecting the step of pyrophosphate removal at each catalytic cycle of DNA polymerization (Zhang et al., 2016). In the presence of Zn, human DNA polymerase α could catalyze the nucleotidyltransfer in a non-template way, although the sugar moiety of the incorporated nucleotide seemed distorted or otherwise cleaved (Sirover and Loeb, 1976). These results and the Zn-induced *in-vitro* DNA replication stress demonstrated in this study reminded that the potential genetic toxicity of Zn should not be ignored while Zn was used as health supplement additive. Previous studies have found the Cr-impeded DNA replication *in vitro* by DNA polymerase Taq, E.Coli Pol I, and mammalian Pol α and β (Bridgewater et al., 1998), which might be interpreted as the formation of Cr-DNA adducts, Cr-DNA inter-strand cross-links, and Cr-induced polymerase arresting lesions (O'Brien et al., 2002). Additionally, EDTA significantly decreased the Cr-DNA binding and the formation of Cr-induced polymerase arresting lesions (O'Brien et al., 2002). In the current study, the rate-limiting effect on DNA replication by Co, Mn, and Ni to a lesser extent was observed at relatively higher metal concentrations. This was probably because these three HMs could serve as cofactors with DNA polymerase for the nucleotidyltransfer reaction, although the substitution of them reduced the exonuclease activity of the enzyme and contributed to the frequency of incorrect dNTPs incorporation (Snow et al., 1993, Tabor and C.C.R. , 1989, Vashishtha and Konigsberg, 2016). Additionally, the latest list of carcinogens in 2023 published by the IARC (https://www.iarc.fr/) and classified Cu at IARC Group 3 (not classifiable as to its carcinogenicity to humans) while Soluble Co(II) and Pb at IARC Group 2A (probable) and 2B (possible) carcinogens, which were consistent with the stress of these HMs induced DNA replication demonstrated in this study. The HMs-induced DNA replication stresses demonstrated here were partly in agreement with previous studies researching HMs-induced diseases related to DNA replication at different levels. These results suggested that the HMs-induced DNA replication stress might play a significant role in HMs-induced DNA replication associated diseases including cancer and developmental disorders.

DNA replication is a complex and precise progress involving stages of initiation, elongation, and termination in prokaryotes and eukaryotes. More than one DNA polymerase is needed in DNA replication, such as polymerase α, β, γ, δ, and ε in eukaryotes, polymerase Ⅰ, Ⅱ, Ⅲ and Taq polymerase in prokaryotes (Weaver, 2011). Despite the differences in detailed domain structures, the polymerases were found to share a common overall architectural feature, which has been described as “thumb,” “palm,” and “fingers” domains, and use the similar oriented secondary structural elements (Kohlstaedt et al., 1992, Steitz, 1999). Additionally, though the polymerases of eubacteria and eukaryotes are not homologous (Bailey et al., 2006), the crystal structures of different polymerases show that all polymerases are very likely to use the same ion-dependent mechanisms to catalyze the polymerase phosphoryl transfer reactions (Gao and Yang, 2016, Steitz, 1993, Yang et al., 2016). Therefore, the results obtained in the current study still provided significant evidence explaining molecular mechanisms of the DNA replication stresses induced by HMs. However, further investigations are needed to explore the HMs-induced DNA replication stress using DNA polymerases at the molecular level.

In summary, this study demonstrated the HMs-induced DNA replication stress characterized by limited-rate using an *in-vitro* DNA replication system at the molecular level. Our results partly revealed the effect of HMs themselves on DNA replication, which provided new potential mechanisms for explaining HMs-induced genetic and cancer risk.

## CRediT authorship contribution statement

**Qidong Ren:** Investigation, Methodology, Formal analysis, Visualization, Writing – original draft. **Xuejun Guo:** Conceptualization, Methodology, Writing – review & editing, Supervision, Funding acquisition. **Dong Yang:** Supervision, Writing – review & editing. **Chuanfang Zhao:** Writing – review & editing. **Xiangyuan Zhang:** Investigation, Writing – review & editing. **Xinghui Xia:** Writing – review & editing.

## Declaration of competing interest

The authors declare that they have no known competing financial interests or personal relationships that could have appeared to influence the work reported in this paper.

## Data Availability

Data will be made available on request.
