# Supplementary material for: A wide survey of heavy metals-induced in-vitro DNA replication stress characterized by rate-limited replication
Source: Curr Res Toxicol. 2024 Feb 1;6:100152. doi: 10.1016/j.crtox.2024.100152 (PMC10848000; doi:10.1016/j.crtox.2024.100152)
Supplement: Supplementary data 1 [file mmc1.docx]

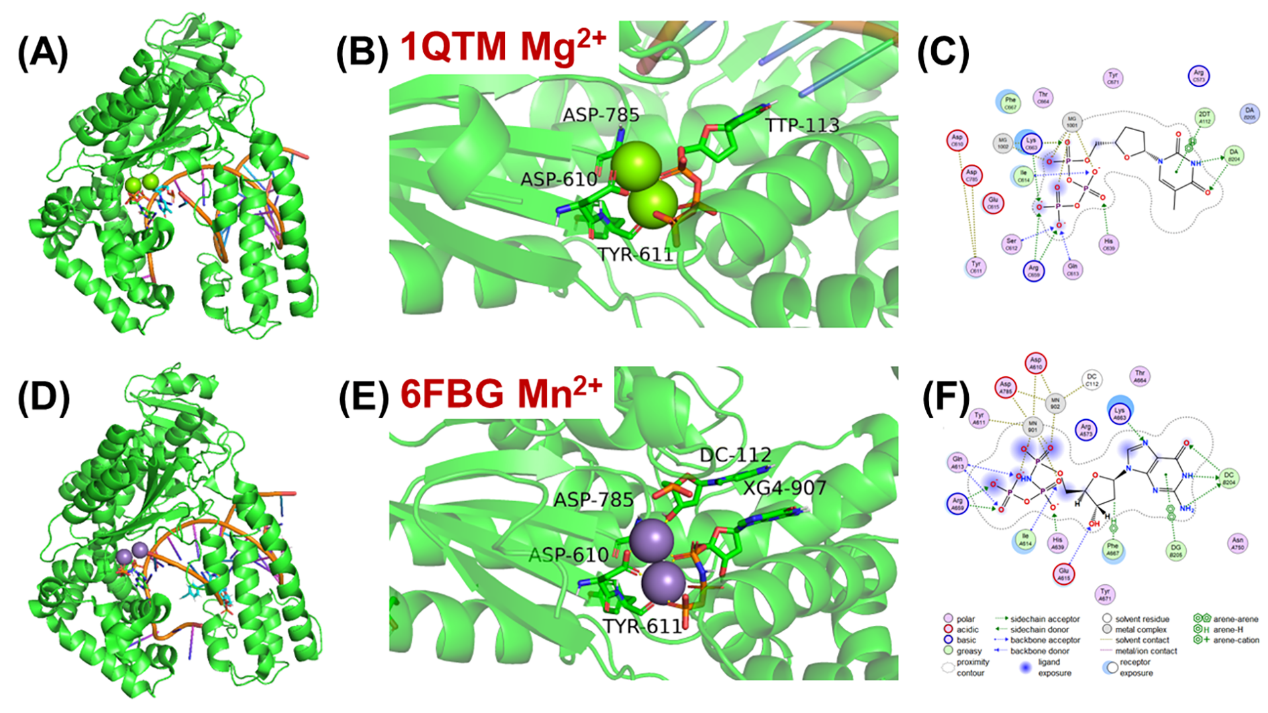


**Figure S1. Structure of Taq DNA polymerase binding with Mg^2+^ and Mn^2+^.** The Taq DNA polymerase catalytic center conformation and binding residues (ASP-785, ASP-610, and TYR-611) of Mg^2+^ (A)-(C) and Mn^2+^ (D)-(F).


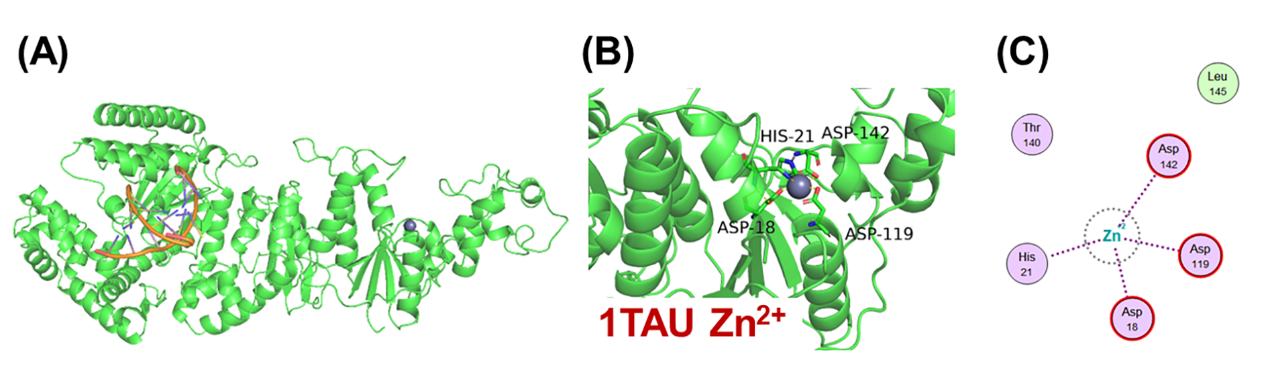


**Figure S2. Structure of Taq DNA polymerase binding with Zn^2+^.** The Taq DNA polymerase catalytic center conformation and binding residues (HIS-21, ASP-18, ASP-119, and ASP-142) of Zn^2+^ (A)-(C).
